# Adaptations in Evoked Pain Sensitivity and Conditioned Pain Modulation after Development of Chronic Neck Pain

**DOI:** 10.1155/2017/8985398

**Published:** 2017-04-06

**Authors:** Bahar Shahidi, Katrina S. Maluf

**Affiliations:** ^1^Physical Therapy and Rehabilitation Science Programs, Department of Physical Medicine and Rehabilitation, University of Colorado Anschutz Medical Campus, Aurora, CO, USA; ^2^Departments of Radiology and Orthopedic Surgery, University of California San Diego, San Diego, CA, USA; ^3^Physical Therapy Program, School of Exercise and Nutritional Sciences, San Diego State University, San Diego, CA, USA

## Abstract

Numerous studies demonstrate elevated pain sensitivity and impaired conditioned pain modulation (CPM) in patients with chronic musculoskeletal pain compared to healthy individuals; however, the time course of changes in pain sensitivity and CPM after the development of a chronic pain condition is unclear. Secondary analysis of data from a prospective investigation examined changes in evoked pain sensitivity and CPM before and after development of chronic neck pain (CNP). 171 healthy office workers participated in a baseline assessment, followed by monthly online questionnaires to identify those who developed CNP over the subsequent year. These individuals (*N* = 17) and a cohort of participants (*N* = 10) who remained pain-free during the follow-up period returned for a 12-month follow-up assessment of mechanical and thermal pain sensitivity and CPM. Pain sensitivity measures did not differ between groups at baseline; however, cold pain threshold decreased in the CNP group at follow-up (*p* < 0.05). CPM was lower at baseline in the CNP group compared to those who reported no neck pain (*p* < 0.02) and remained unchanged one year later. These findings indicate that CPM is reduced in healthy individuals prior to the development of chronic neck pain and the subsequent reduction of thresholds for cold but not pressure pain.

## 1. Introduction

Quantitative sensory testing (QST) is a commonly utilized and feasible method of assessing pain sensitivity and pain modulatory pathways in both healthy and clinical pain populations [1]. Alterations in the sensitivity to noxious thermal and mechanical stimuli are commonly observed among individuals with acute and chronic pain [2–5]. These alterations are typically measured as changes in the threshold or tolerance for experimentally evoked pain. Conditioned pain modulation (CPM) can assess endogenous pain inhibition using a variety of protocols [6, 7]. CPM is thought to activate descending spinobulbospinal circuits that cause a reduction in sensitivity to a painful phasic (i.e., test) stimulus while experiencing another painful tonic (i.e., conditioning) stimulus [7, 8]. These sensory assessments provide an indirect estimate of the sensitivity and modulatory efficiency of central and peripheral nervous system processes, which are thought to play a role in the development and persistence of pain [9].

Cross-sectional studies have demonstrated that individuals with chronic neck pain have alterations in both pain sensitivity and CPM compared to those without pain [3, 10]; however, causal inferences and the time course of changes in experimental pain measures cannot be determined from these studies. We recently reported the results of a prospective investigation which found that preexisting impairments in CPM, but not pain sensitivity, increased the risk of developing chronic neck pain in otherwise healthy individuals [11]. This finding is in agreement with another recent prospective investigation of healthy individuals which also found that mechanical pain sensitivity failed to predict the future development of temporomandibular pain [12]. However, CPM was not investigated in the latter study, so it is still not known how impairments in CPM develop and change over time in healthy people who develop chronic musculoskeletal pain.

Characterizing the temporal sequence of adaptations in pain sensitivity and pain modulation before and after the transition to chronic pain may help practitioners recognize early signs of central sensitization and intervene at appropriate time points. Therefore, we conducted an exploratory secondary analysis of data from our prospective investigation of risk factors for chronic neck pain to determine if changes in pain sensitivity and CPM differed for individuals who did and did not develop chronic neck pain during their first year of employment in a high-risk occupation. We hypothesized that impairments in CPM would be observed prior to increases in thermal and mechanical pain sensitivity in individuals who developed chronic neck pain, whereas CPM and pain sensitivity would not change in those who remained pain-free throughout the 12-month follow-up.

## 2. Materials and Methods

### 2.1. Study Participants

Participants included in this exploratory analysis were recruited from 171 healthy office workers enrolled in a prospective study [11] conducted from 2011 to 2014 at the University of Colorado Anschutz Medical Campus. Briefly, participants included healthy office workers who were 18 to 65 years of age and within 3 months of their date of hire in a new job that required them to work ≥30 hours per week in an office setting, with the use of a computer for at least 75% of the workday. Participants were eligible for inclusion if they reported no neck pain or neck-related disorders during the previous year, scored <5 points on the Neck Disability Index (NDI), and demonstrated no signs of cervical pathology during a standardized physical examination [13]. Individuals with a current injury or any prior history of chronic pain and those with a diagnosed medical condition that could affect sensory or motor function were excluded from participation. 171 enrolled participants completed a baseline assessment and were followed prospectively for 12 months through administration of a monthly online survey (REDCap Software, v 5.5.9, Vanderbilt University, 2014) to identify those who developed chronic interfering neck pain. Based on published recommendations from the Task Force on Neck Pain [14], chronic interfering neck pain was operationally defined as the self-reported presence of neck-related activity limitations (indicated by NDI scores ≥ 5 points) and/or health care utilization for 3 or more months during the 12-month follow-up period [11]. Neck pain was considered to be chronic if present and limiting for at least 3 months during the past year, regardless of whether the pain was persistent or intermittent in nature.

Of the 171 healthy office workers who completed a baseline assessment of pain sensitivity and CPM in our previous investigation, one was excluded due to a neck injury sustained in a motor vehicle accident and 3 others failed to complete at least 8 of 12 neck disability surveys administered during the 12-month follow-up. Of the remaining participants, 35 individuals developed chronic interfering neck pain and were invited to return for a follow-up assessment of primary measures of pain sensitivity and CPM for the present investigation. A comparison group of 29 individuals who reported a complete absence of neck pain and neck-related disability during follow-up (NDI = 0 points for 12 consecutive months) were also invited to return for a follow-up assessment. Participants who reported low levels of intermittent neck disability (NDI = 1 to 4 points) over the course of the year that did not meet threshold criteria for chronic interfering neck pain were not invited for follow-up.

### 2.2. Procedures for Assessment of Pain Sensitivity and CPM

A baseline assessment of pain sensitivity, CPM, age, sex, body mass index (BMI), current neck pain (10 cm Visual Analog Scale for pain intensity; VAS), current neck disability (NDI), and history of prior injuries was conducted within the first 3 months of hire into a high-risk occupation for all enrolled participants. Primary assessments of pain sensitivity and CPM were repeated during a follow-up session conducted approximately 12 months after the baseline assessment for participants who either developed chronic neck pain or remained pain-free during the observation period. Current levels of neck pain (VAS) and disability (NDI) were also assessed on the day of testing at follow-up; however, the examiner remained blind to neck pain status during testing. For all assessments involving quantitative sensory testing, both the participant and the assessor were blind to force readings, which were digitally recorded and analyzed offline by a research assistant who was blind to neck disability status.

Evaluation of pain sensitivity included responses to noxious thermal and mechanical stimuli. Cold pain threshold (CPThr) and cold pain tolerance (CPTol) were quantified as the amount of time in seconds required for a cold sensation to be perceived as slightly painful and intolerable, respectively, during submersion of the nondominant hand into a 4°C circulating water bath to wrist level (i.e., cold pressor test). Mechanical pain sensitivity was measured using a digital pressure algometer (Wagner Instruments, Greenwich, CT) with a 1 cm diameter rubber tip manually applied at a rate of 1 kgF/s over the dominant upper trapezius muscle belly and 2 cm lateral to the midpoint between the seventh cervical vertebrae and the acromion process. Pressure pain threshold (PPT) was defined as the lowest pressure at which the subject verbally indicated that the sensation of pressure was first perceived as slightly painful. PPT scores comprised an average of 3 trials, separated by at least 60-second rest between trials [15].

CPM assessed endogenous pain inhibition by measuring the change in PPT for the dominant upper trapezius muscle (phasic test stimulus) during submersion of the nondominant hand in a 4°C circulating water bath (tonic conditioning stimulus) compared with a circulating water bath maintained at room temperature to control for the nonthermal sensory effects of water immersion. The two temperature conditions were randomized, with at least 30 minutes between conditions to control for any residual analgesic effects of the cold pressor test. The PPT test stimulus was applied immediately after the CPThr was reached during the cold pressor test and 30 seconds after immersion of the hand in room temperature water in the control condition. The average of 3 PPT trials collected at 30-second intervals was calculated for each temperature condition, with fewer trials included in the average for participants who reached CPTol before 3 PPT trials could be collected during the cold pressor test. The change in PPT between temperature conditions was expressed relative to the control (room temperature) condition according to the following formula: %  CPM = [(PPT_cold_ − PPT_control_)/PPT_control_]*∗*100, where higher values indicate more efficient pain inhibition.

### 2.3. Data Analysis

Demographic and clinical characteristics were compared between groups using independent *t*-tests for continuous variables and chi-square tests for nominal variables. Histograms for all variables were checked for normality using skewness (±0.8) and kurtosis (±2) thresholds [16]. To identify impairments in pain processing at baseline, group differences were assessed using the Mann-Whitney* U* test for nonparametric data (PPT, CPThr) and independent *t*-tests for parametric data (CPM, CPTol). A priori hypotheses regarding changes in pain processing over time were then assessed separately for each group using the Wilcoxon Signed Rank Test for nonparametric data and paired *t*-tests for parametric data. Due to the small sample size available for analysis, effect sizes were calculated to estimate the magnitude of differences. All statistical analyses were performed using SAS statistical software (SAS statistical software v 9.3.3, Cary, North Carolina). Values are reported as mean (SD) in the text and figures unless otherwise indicated.

## 3. Results

**Figure 1:**
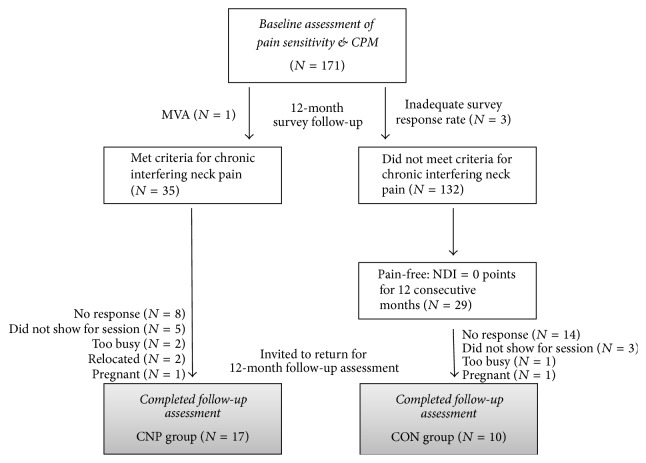
Flow diagram for participant enrollment and dropouts. CPM = conditioned pain modulation; MVA = motor vehicle accident; NDI = Neck Disability Index; CNP = chronic neck pain; CON = control.

Thirty-five participants with chronic interfering neck pain and 29 participants who remained pain-free were invited to participate in the 12-month follow-up assessment. Of these, 17 participants with chronic neck pain (CNP) and 10 pain-free controls (CON) returned for testing. The reasons for dropout are provided in the enrollment diagram (Figure 1). Comparison of baseline characteristics including age, sex, BMI, neck disability, and those reporting a prior history of musculoskeletal injury revealed no significant differences between those who did and did not return to complete the follow-up assessment (Table 1). NDI scores averaged over the 12-month follow-up period also did not differ between participants with CNP who did and did not complete the follow-up assessment (4.2 (1.7) versus 4.4 (1.6) points;* p* = 0.65). Similarly, baseline characteristics did not differ between completers in the CNP and CON groups (Table 1, Italic values;* p* ≥ 0.51). Participants in the CNP group had an average pain intensity rating of 1.62 (0.69) cm on the day of follow-up testing, whereas all participants in the CON group reported a VAS score of 0 cm.

**Figure 2:**
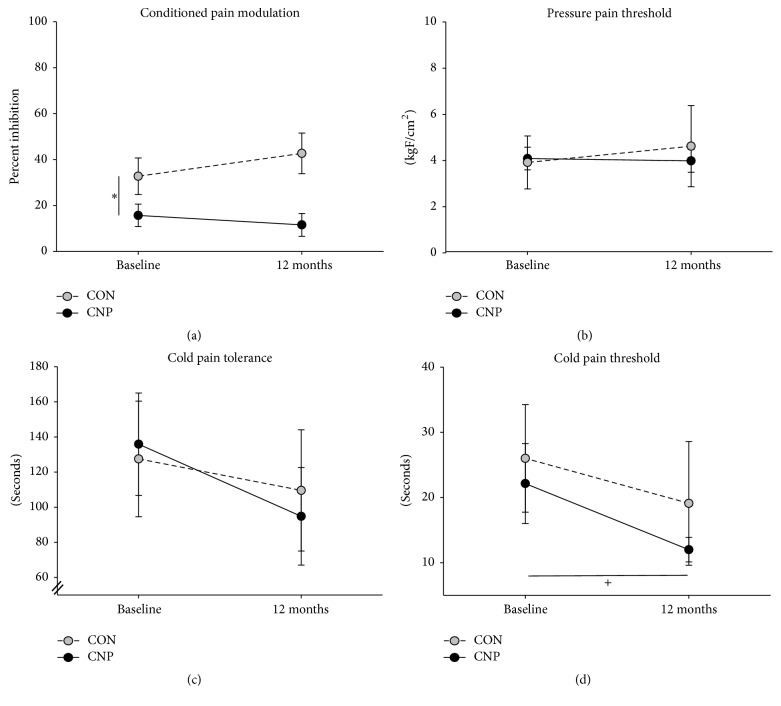
Changes in conditioned pain modulation (a), pressure pain threshold (b), cold pain tolerance (c), and cold pain threshold (d) from baseline to 12-month follow-up assessment for the group who developed chronic neck pain (CNP; black circles) and a control group who remained pain-free (CON; gray circles). Values are mean (SD). ^*∗*^*p* = 0.05 between groups at baseline. ^+^*p* = 0.02 from baseline to 12 months for CNP group.

Results for CPM and pain sensitivity outcomes are illustrated in Figure 2. CPM was lower at baseline for the CNP group compared to the CON group (*t*_(25)_ = 2.0,* p* = 0.05, Cohen's* d* = 0.80). There were no group differences at baseline for PPT (*U* = 48.0,* p* > 0.05), CPThr (*U* = 77.5,* p* > 0.05) or CPTol (*t*_(25)_ = −0.02,* p* > 0.05). Participants in the CNP group demonstrated significant decreases in CPThr over time (*Z* = −2.42,* p* = 0.02, and* r* = 0.40), whereas CPThr did not change significantly in the CON group (*Z* = −1.93,* p* > 0.05). A similar but nonsignificant trend was observed for CPTol, with a decrease in pain tolerance over time for the CNP group (*t*_(16)_ = 1.96,* p* = 0.07, and Cohen's* d* = 0.48) but not the CON group (*t*_(9)_ = −0.08,* p* > 0.05). There were no significant changes in PPT over time for either the CNP group (*Z* = 0.00,* p* > 0.05) or the CON group (*Z* = −0.77,* p* > 0.05).

## 4. Discussion

The results of this exploratory analysis indicated that healthy individuals at risk of chronic neck pain who had* preexisting* impairments in CPM did not demonstrate further changes in CPM after a chronic pain condition developed. In contrast, thermal pain thresholds were similar between groups at baseline and decreased significantly only for those who developed chronic neck pain, whereas mechanical pain thresholds showed no differences over time or between groups. Together with our previous findings [11], these results suggest that stable impairments in endogenous pain inhibition increase the risk for chronic neck pain in healthy individuals and that early central nervous system adaptations to chronic pain may be manifested primarily through a reduction in thermal but not mechanical pain thresholds.

Many studies have observed reduced endogenous pain inhibition among patients with chronic musculoskeletal pain, whereas other studies have reported that CPM is preserved. Impaired CPM has been shown to predict minor episodes of acute pain and reduced physical function even among healthy individuals [17]. However, few prospective studies have investigated whether alterations in CPM occur prior to or as a consequence of chronic pain in formerly healthy individuals or whether these impairments continue to worsen once a chronic condition has developed. Existing studies have demonstrated associations between CPM, evoked pain sensitivity, and pre- and postoperative pain intensity in clinical pain populations. For example, Yarnitsky et al. found that individuals with lower preoperative CPM were more likely to develop chronic postoperative pain when undergoing surgical thoracotomy [18]. Similarly, Kosek and Ordeberg, observed recovery of impaired CPM in individuals with chronic hip osteoarthritis after surgery [19], whereas patients with knee osteoarthritis who had impaired CPM and facilitated temporal summation of pain were less likely to experience pain relief after total knee replacement surgery [20]. To our knowledge, the current study is the first to measure CPM before and after the development of chronic pain in otherwise healthy individuals. Our findings demonstrate that impairments in CPM are present prior to the onset of chronic neck pain and precede subsequent changes in evoked cold pain threshold. Furthermore, CPM appears to remain stable in the early stages of chronicity (i.e., within the first year of relatively mild persistent or intermittent neck pain).

Evoked pain sensitivity has been widely investigated in cross-sectional studies of individuals with and without chronic pain, and the majority of evidence suggests that reductions in the threshold and tolerance for noxious thermal and mechanical stimuli (i.e., increases in pain sensitivity) are a common feature of chronic pain conditions [2–5]. Although individuals with existing pain show greater sensitivity to evoked thermal and mechanical stimuli, thermal pain sensitivity seems to have greater predictive value for the future development of musculoskeletal pain [12, 21]. This is in contrast to our previous finding that neither thermal nor mechanical pain sensitivity was able to predict the development of chronic neck pain [11]; however, the present study demonstrates that thermal pain thresholds decrease early after the onset of neck pain, whereas mechanical pain thresholds do not. Thus, early changes in thermal pain sensitivity following new onset chronic neck pain in the absence of corresponding changes in mechanical pain sensitivity may indicate a distinct time course for adaptations in processing of different sensory modalities. This novel observation may inform future efforts to identify early indicators of the transition to chronic pain. The hypothesis that thermoreceptors may demonstrate faster adaptations to persistent pain than mechanoreceptors is supported by evidence of thermal allodynia within 1 day of experimentally induced injury in non-primate animals, whereas mechanical allodynia either developed gradually up to 30 days after injury or was not present [22, 23]. Differences between thermal and mechanical pain thresholds observed in the present study indicate a need for longitudinal studies to examine the time course of changes in evoked pain sensitivity for various sensory modalities in the transition from acute to chronic pain.

The primary limitation of this exploratory study was the small sample of participants with baseline assessments of pain sensitivity and CPM who volunteered to return for a follow-up assessment one year later. Although we were able to demonstrate significant changes in cold pain threshold after the development of CNP, the current analysis lacks power to detect more subtle changes in pain processing that may occur over time in individuals with and without chronic neck pain. Despite limitations in power, prospective data of this nature remain widely unavailable in the literature and provide novel information that can inform future investigations of the time course of changes in pain sensitivity and modulation during the transition to chronic pain. Specifically, this is the first study to measure adaptations in pain sensitivity and CPM measures over an extended period of time (i.e., longer than a few weeks [6]) in pain-free individuals and to report the temporal sequence of adaptations in these measures before and after the development of a* chronic* pain condition. Although comparisons between those who did and did not complete the 12-month follow-up assessment revealed no obvious selection bias, our findings should be considered preliminary until reproduced in larger samples. Furthermore, our CNP sample included those with both persistent and intermittent symptoms so it is unclear to what extent adaptations in pain processing may differ in timing or magnitude for these clinically distinct subgroups.

## 5. Conclusions and Future Directions

Changes in thermal pain thresholds after the development of chronic neck pain are preceded by impairments in CPM which are present before the onset of pain. Endogenous pain inhibition predisposes development of chronic neck pain in healthy individuals whereas evoked pain sensitivity does not, despite the decrease in thermal pain threshold that emerges in the early stages of a persistent pain condition. The ability to quantify preexisting impairments in CPM may help identify susceptible individuals for primary prevention of chronic neck pain. Similarly, distinct temporal trajectories for adaptations in cold and pressure pain sensitivity may prove useful in recognizing early signs of central sensitization in the transition from acute to chronic pain.

## Figures and Tables

**Table 1:** Comparison of baseline characteristics for participants with chronic neck pain (CNP) and pain-free controls (CON) who did and did not return to complete the 12-month follow-up assessment.

	CNP completers (*N* = 17)	CNP dropouts (*N* = 18)	*p*	CON completers (*N* = 10)	CON dropouts (*N* = 19)	*p*
Age (years)	*27.9 (7.0)*	31.2 (7.5)	0.19	*26.3 (3.3)*	20.0 (9.2)	0.38
Sex (M : F)	*3 : 14*	1 : 17	0.34	*1 : 9*	7 : 12	0.12
BMI (kg/m^2^)	*23.6 (4.8)*	23.6 (3.2)	1.0	*23.0 (3.7)*	25.0 (3.9)	0.19
NDI (points)	*0.69 (1.14)*	1.1 (1.30)	0.33	*0.50 (1.00)*	0.10 (0.23)	0.10
Prior injury (%)	*18.5*	33.3	0.44	*11.1*	36.8	0.20
